# Relationship between Carotenoids, Retinol, and Estradiol Levels in Older Women

**DOI:** 10.3390/nu7085296

**Published:** 2015-08-05

**Authors:** Marcello Maggio, Francesca de Vita, Fulvio Lauretani, Stefania Bandinelli, Richard D. Semba, Benedetta Bartali, Antonio Cherubini, Anne R. Cappola, Gian Paolo Ceda, Luigi Ferrucci

**Affiliations:** 1 Department of Clinical and Experimental Medicine, University of Parma, Via Gramsci 14, Parma 43126, Italy; E-Mails: marcellomaggio2001@yahoo.it (M.M.); gpceda@unipr.it (G.P.C.); 2 Geriatric Rehabilitation Department, University Hospital of Parma, Via Gramsci 14, Parma 43126, Italy; E-Mail: flauretani@ao.pr.it; 3 Geriatric Unit, Azienda Sanitaria di Firenze, Florence 50100, Italy; E-Mail: stefania.bandinelli@asf.toscana.it; 4 Department of Ophthalmology, Johns Hopkins University School of Medicine, 733 North Broadway Baltimore, MD 21225, USA; E-Mail: rdsemba@jhmi.edu; 5 New England Research Institute, 480 Pleasant Street, Watertown, MA 02472, USA; E-Mail: bbartali@neriscience.com; 6 Geriatrics, IRCCS-INRCA, Via della Montagnola, Ancona 81 60127, Italy; E-Mail: A.CHERUBINI@inrca.it; 7 Division of Endocrinology, Diabetes, and Metabolism, Department of Medicine, Perelman School of Medicine at the University of Pennsylvania, 295 John Morgan Building, 3620 Hamilton Walk, Philadelphia, PA 19104, USA; E-Mail: acappola@mail.med.upenn.edu; 8 National Institute on Aging, National Institutes of Health (NIH), Harbor Hospital 3001 Hanover Street Baltimore, MD 21225, USA; E-Mail: ferruccilu@grc.nia.nih.gov

**Keywords:** estrogens, carotenoids, retinol, elderly, women

## Abstract

Background. *In vitro* evidence suggests anti-estrogenic properties for retinol and carotenoids, supporting a chemo-preventive role of these phytochemicals in estrogen-dependent cancers. During aging there are significant reductions in retinol and carotenoid concentrations, whereas estradiol levels decline during menopause and progressively increase from the age of 65. We aimed to investigate the hypothesis of a potential relationship between circulating levels of retinol, carotenoids, and estradiol (E2) in a cohort of late post-menopausal women. Methods. We examined 512 women ≥ 65 years from the InCHIANTI study. Retinol, α-caroten, β-caroten, β-criptoxantin, lutein, zeaxanthin, and lycopene levels were assayed at enrollment (1998–2000) by High-Performance Liquid Chromatography. Estradiol and testosterone (T) levels were assessed by Radioimmunometry (RIA) and testosterone-to-estradiol ratio (T/E2), as a proxy of aromatase activity, was also calculated. General linear models adjusted for age (Model 1) and further adjusted for other confounders including Body Mass Index (BMI) BMI, smoking, intake of energy, lipids, and vitamin A; C-Reactive Protein, insulin, total cholesterol, liver function, and testosterone (Model 2) were used to investigate the relationship between retinol, carotenoids, and E2 levels. To address the independent relationship between carotenoids and E2 levels, factors significantly associated with E2 in Model 2 were also included in a fully adjusted Model 3. Results. After adjustment for age, α-carotene (β ± SE = −0.01 ± 0.004, *p* = 0.02) and β-carotene (β ± SE = −0.07 ± 0.02, *p* = 0.0007) were significantly and inversely associated with E2 levels. α-Carotene was also significantly and positively associated with T/E2 ratio (β ± SE = 0.07 ± 0.03, *p* = 0.01). After adjustment for other confounders (Model 2), the inverse relationship between α-carotene (β ± SE = −1.59 ± 0.61, *p* = 0.01), β-carotene (β ± SE = −0.29 ± 0.08, *p* = 0.0009), and E2 persisted whereas the relationship between α-carotene and T/E2 ratio was attenuated (β ± SE = 0.22 ± 0.12, *p* = 0.07). In a fully adjusted model (Model 3), only β-carotene (β ± SE = −0.05 ± 0.02, *p* = 0.03) was significantly and inversely associated with E2 levels independent of α-carotene. No association was found between retinol, total non-pro-vitamin A carotenoids, lutein, zeaxanthin, and lycopene, and E2 levels. Conclusions: In older women, β-carotene levels are independently and inversely associated with E2.

## 1. Introduction

Carotenoids comprise a class of natural hydrocarbons (carotenes) and their oxygenated derivatives (xanthophylls) that are important components of the antioxidant network [1]. Some of these phyto-nutrients (*i.e.*, β-carotene, α-carotene, and β-cryptoxanthin) undergo intestinal bio-conversion into retinol and retinyl esters by the 15-15′-dioxygenase, functioning as the major dietary sources of vitamin A needs in humans [2].

Epidemiological studies suggest an inverse association between dietary consumption of plant products rich in carotenoids and vitamin A (retinol), such as leafy greens and yellow-colored vegetables and orange-colored fruit, and the incidence of various types of human cancer [3]. The chemo-protective action of these phytochemicals includes antioxidant properties, modulator activities on cell proliferation, growth factor signaling, and gap junctional intercellular communication [4].

Interestingly, recent *in vitro* studies indicate that carotenoids and retinol also exert a beneficial role in the prevention of estrogen receptor (ER)-positive cancers, such as mammary and endometrial [5,6,7,8,9,10,11], through the inhibition of either the activity of 17 β-estradiol (E2 or aromatase enzyme. Approximately 70% of breast cancers in women requires estrogens for cell proliferation and survival, and the effect of E2 on target cells is almost exclusively mediated by its binding to the estrogen receptor-α (ERα) [8].

Anti-estrogenic properties have been also documented for β-carotene and the major dietary non-provitamin A carotenoid lycopene [12]. In breast and endometrial cancer cells, β-carotene and lycopene inhibit cancer cell proliferation induced by E2 [13], also attenuating the DNA damage caused by catechol-estrogens [14]. A nested case-control study using plasma collected from women enrolled in the Nurses’ Health Study showed that breast cancer is 25%–35% less frequent in those postmenopausal women with the highest quintile of α-carotene, β-carotene, and lutein/zeaxanthin compared with that for women in the lowest quintiles [15]. Other studies demonstrated a correlation between a diet rich in carotenoids (α-carotene and β-carotene [16] and lycopene [17]), and a lower risk for breast and endometrial cancers. Interestingly, such an inverse association was stronger for post-menopausal women with estrogen receptor (ER)-positive cancers [13].

Still, E2 levels decline during menopause, with a trend of increasing E2 levels from the age of 65 years onward [18]. The opposite phenomenon has been observed for retinol and carotenoid plasma concentrations, whose levels undergo a significant decline with aging [19,20].

However, the hypothesis of a potential relationship between circulating levels of retinol, carotenoids, and E2 has never been addressed in the older female population. Therefore, we investigated whether retinol, pro-vitamin (β-carotene, α-carotene, and β-cryptoxanthin), and non-provitamin A carotenoid (lycopene, zeaxanthin, and lutein) concentrations were significantly associated with E2 levels in a cohort of Italian late post-menopausal women not taking hormone replacement therapy (HRT).

## 2. Subjects and Methods

### 2.1. Subjects

InCHIANTI is an epidemiological study of risk factors for mobility disability in the elderly, designed by the Laboratory of Clinical Epidemiology of the Italian Research Council of Aging (Florence) and conducted on a representative sample of a population living in Greve in Chianti and Bagno a Ripoli, two small towns in the Chianti geographic area (Tuscany, Italy). The initial population was composed of 1453 participants (aged 22–104) randomly selected from the residents of these two municipalities using a multistage stratified sampling method. Baseline data collection started in September 1998 and was completed in March 2000. The rationale, design, and data collection have been described elsewhere, and the main outcome of this longitudinal study is mobility disability [21]. Of the whole study population, 1353 donated a blood sample and 1063 subjects were aged 65 years or older. Of those older individuals, 545 were women with complete data on total E2, T, retinol, α-carotene, β-carotene, β-criptoxantin, lutein, zeaxanthin, and lycopene. After the exclusion of 33 women who were using oral HRT, 512 women were selected for the study presented here. Of this subset of participants, 450 had natural menopause and 62 had surgical menopause. None of the participants were taking multivitamins containing vitamin A or androgens.

The Italian National Institute of Research and Care on Aging Institutional Review Board approved the study protocol. Participants consented to participate and to have their blood samples analyzed for scientific purposes.

### 2.2. Biological Samples

Blood samples were collected and analyzed at baseline from participants after a 12 h fast and after a 15 min rest. Aliquots of serum were stored at −80 °C and were not thawed until analyzed.

### 2.3. Laboratory Measures

#### 2.3.1. Retinol and Carotenoids (Predictors)

Measures for carotenoid levels were obtained from frozen plasma samples originally collected at baseline. Aliquots of plasma were shipped to the laboratory of the Johns Hopkins University (Baltimore, MD, USA), on dry ice for measurements of plasma carotenoids.

α-Carotene, β-carotene, β-cryptoxanthin, lutein, zeaxanthin, and lycopene were measured using high-performance liquid chromatography (HPLC). The laboratory protocol was developed by Schleicher at the Centers for Disease Control in Atlanta and is described in better detail in the paper by Dorgan [22].

Total carotenoids were calculated as the sum of α-carotene, β-carotene, β-cryptoxanthin, lutein, zeaxanthin, and lycopene in micromoles per liter (μmol/L). Within-run and between-run coefficients of variation (CVs) were 7.3% and 9.6% for α-carotene, 4.5% and 5.4% for β-carotene, 2.7% and 3.5% for β-cryptoxanthin, 2.6% and 7.1% for lutein, 6.2% and 6.8% for zeaxanthin, and 7.5% and 7.8% for lycopene, respectively.

Plasma retinol was measured using an isocratic high-performance liquid chromatography method as described by Sowell *et al.* [23]. Retinol was isolated using a 150 × 4.6 mm octadecylsilane column packed with 5 μm particles with a mobile phase of an equivolume solution of ethanol and acetonitrile containing 0.1 mL of diethylamine per liter of solvent. The flow rate was 0.9 mL/min and the concentration was determined at 325 nm wavelength. The within-run and between-run coefficients of variation were 3.3% and 2.8%.

#### 2.3.2. Hormone Assays (Outcomes)

Estradiol levels were measured in the Laboratory of the University of Parma (Parma, Italy) using ultrasensitive radioimmunoassay (RIA) using a DSL-4800 Kit (Diagnostic Systems Laboratories, Webster, TX, USA) with a minimum detectable concentration (MDC) of 2.2 pg/mL. Intra-assay CVs and means for four different concentrations were 8.9% (5.3 pg/mL), 6.5% (24.9 pg/mL), 7.6% (40.4 pg/mL), and 6.9% (92.6 pg/mL). The inter-assay CVs and correspondent means were 7.5% (5.3 pg/mL), 9.7% (28.0 pg/mL), 8.0% (42.3 pg/mL), and 12.2% (108.7 pg/mL), respectively.

Testosterone was assayed using commercial radioimmunological kits (Diagnostic Systems Laboratories, Webster, TX, USA). The MDC was 0.08 nmol/L; intraassay and interassay CVs for three different concentrations were 9.6%, 8.1%, and 7.8%, and 8.6%, 9.1%, and 8.4%, respectively.

The testosterone-to-estradiol ratio (T/E_2_) was calculated as a proxy for aromatase enzymatic activity.

### 2.4. Other Measures

Data from healthy elderly participants of the Framingham Heart Study [24] have shown that important correlates of most carotenoids include body mass index (negative effect), smoking status (negative effect), and plasma cholesterol concentration (negative effect). Thus, these factors were all considered as possible confounders of the association between carotenoids, retinol, and E2 levels.

Weight was measured to the nearest 0.1 kg using a high-precision mechanical scale, with the participant wearing light clothes and without shoes. Standing height without shoes was measured to the nearest 0.1 cm. Body mass index was calculated as (weight (kg))/(height (m)^2^) and obesity was defined as BMI > 30 kg/m^2^.

Smoking history was determined based on self-report and participants were categorized into never smokers, former smokers, and current smokers.

Plasma lipid levels were measured at baseline on serum from fresh samples drawn after 12 h overnight fasting. Commercial enzymatic tests (Roche Diagnostics, Basel, Switzerland) were used for determining serum total cholesterol, triglycerides, and High Density Lipoprotein (HDL)-Cholesterol(C) concentrations. The interassay CV was less than 3.8% for total cholesterol and less than 5.0% for HDL-C. For triglycerides, the lower MDC was 4.0 mg/dL, and the intraassay and interassay CVs were 3.1%, and the CV was 1.8%, respectively.

The diagnosis of specific medical conditions was established using standardized criteria that combine information from self-reported history, medical records, and a clinical medical examination. Diagnostic algorithms were modified versions of those created for the Women’s Health and Aging Study [25].

Fasting insulin was determined at baseline on plasma samples stored at −80 °C using a commercial double-antibody, solid-phase radioimmunoassay (Sorin Biomedica, Milan, Italy). Liver function was evaluated by aspartate aminotransferase (AST) and alanine aminotransferase (ALT). Serum creatinine (a marker of kidney function) was measured by a standard creatinine Jaffe method (Roche Diagnostics, Mannheim, Germany).

C-reactive protein (CRP) was measured at baseline with a high-sensitivity enzyme-linked immunosorbent assay (ELISA) a competitive immunoassay that uses purified protein and polyclonal anti-CRP antibodies. The inter-assay CV was 5%. The MDC was 0.03 mg/L.

### 2.5. Nutrient Intake 

Average daily intakes of energy (kcal), carbohydrates, total protein, total lipids, *etc.* were estimated using the European Prospective Investigation into Cancer and Nutrition (EPIC) food frequency questionnaire which has been validated for use in the older population [25].

According to newer research the relative vitamin A activity of β-carotene is only half as much as previously thought [26]. Therefore, as recommended by the Institute of Medicine, vitamin A intake was calculated in Retinol Activity Equivalents (RAE) (retinol (mcg) plus (β-carotene equivalents (mcg)/12)) [27]. β-Carotene equivalents included vitamin A activity from the provitamin A carotenoids: β-carotene, α-carotene, and β-cryptoxanthin. Because the vitamin A activity of α-carotene and β-cryptoxanthin is approximately 50% of β-carotene, β-carotene equivalents (mcg/day) were calculated as β-carotene (mcg) plus one-two (α-carotene (mcg) + β-cryptoxanthin (mcg)) [27].

### 2.6. Statistical Analysis

Variables normally distributed were reported as mean values ± SD and categorical values as the number of cases and percentages. Total carotenoids were categorized into total pro-vitamin A (α-carotene, β-carotene, β-criptoxantin) and total non-provitamin A carotenoids (lutein, zeaxanthin, and lycopene). Because of skewed distribution, median values and interquartile ranges were used as descriptive statistics for total provitamin A carotenoids, total non-provitamin A carotenoids, E2, T, T/E_2_ ratio, insulin, AST, ALT, creatinine, CRP, caloric intake, and RAE. In the current analysis these variables were log-transformed and then back-transformed for data presentation. 

Relations of retinol and carotenoids to E2 levels as well relationships between retinol and carotenoids and the T/E2 ratio, as proxy of the aromatase enzymatic, were tested in an age-adjusted linear regression model (Model 1).

Significant relationships were further adjusted for confounders including BMI, T, insulin, total cholesterol, CRP, smoking, AST, ALT, creatinine, cancer, caloric intake, lipid intake, and RAE (Model 2). Factors significantly associated with E2 in Model 2 were also included in a fully adjusted Model 3, in order to investigate the independent relationship between carotenoids and E2 levels. Statistical significance was defined as *p* < 0.05. SAS 8.2 statistical package was used for all analyses (SAS Institute, Inc., Cary, NC, USA).

## 3. Results

Characteristics of the study population are presented in Table 1. The mean age and standard deviation (SD) of the study sample was 76.3 ± 7.8 years. After adjustment for age (Model 1, Table 1), total carotenoids (β ± SE = −0.11 ± 0.05, *p* = 0.03) and total pro-vitamin A carotenoids (β ± SE = −0.07 ± 0.03, *p* = 0.009) were significantly and negatively associated with E2 levels. Among the pro-vitamin A carotenoids, only α-carotene (β ± SE = −0.01 ± 0.004, *p* = 0.02) and β-carotene (β ± SE = −0.07 ± 0.02, *p* = 0.0007) were significantly and inversely associated with E2 levels. No association was found between β-Cryptoxanthin, retinol, lutein, zeaxanthin, and lycopene, or total non-pro-vitamin A carotenoids and E2 levels.

In Model 2, after adjustment for confounders, including BMI, smoke, caloric, lipid and vitamin A intake (RAE), CRP, insulin, total-cholesterol, liver function, and T, the significant inverse relationship of α-carotene (β ± SE = −1.59 ± 0.61, *p* = 0.01) and β-carotene (β ± SE = −0.29 ± 0.08, *p* = 0.0009) to E2 remained statistically significant (Table 2).

However, in a fully adjusted Model 3 (Table 3) including only the factors significantly associated with E2 in Model 2, β-carotene (β ± SE = −0.23 ± 0.09, *p* = 0.01) but not α-carotene (β ± SE = −0.003 ± 0.003, *p* = 0.35) remained significantly and inversely associated with E2 levels independent of confounders.

In Model 1, α-carotene, but not other carotenoids and retinol, was also significantly and positively associated with the T/E_2_ ratio (β ± SE = 0.07 ± 0.03, *p* = 0.01). However, in the fully adjusted model the relationship between α-carotene and T/E2 was attenuated and no longer statistically significant (β ± SE = 0.22 ± 0.12, *p* = 0.07).

**Table 1:** Characteristics of the study population.

Parameter	All (*N* = 512)
Age (years)	76.3 ± 7.8
BMI (kg/m^2^)	27.7 ± 4.6
Total carotenoids (μmol/L)	1.7 (1.4–2.2)
Total provitamin A carotenoids (μmol/L)	0.7 (0.5–0.9)
α-Carotene (μmol/L)	0.04 (0.03–0.07)
β-Cryptoxanthin (μmol/L)	0.18 (0.11–0.29)
β-Carotene (μmol/L)	0.4 (0.2–0.5)
Total non-provitamin A carotenoids (μmol/L)	1.1 (0.8–1.3)
Lutein (μmol/L)	0.37 (0.26–0.47)
Zeaxanthin (μmol/L)	0.06 (0.04–0.07)
Lycopene (μmol/L)	0.7 (0.4–0.8)
Retinol (μmol/L)	1.8 ± 0.4
Estradiol (pg/mL)	5.6 (3.9–7.8)
Testosterone (nmol/L)	0.6 (0.4–0.8)
Testosterone/Estradiol ratio	0.1 (0.07–0.15)
Insulin (mIU/L)	9.8 (5.0–14.2)
AST (U/L)	19.1 (17.0–22.1)
ALT (U/L)	16.2 (12.2–20.0)
Creatinine (mg/dL)	0.8 (0.7–0.9)
Total Cholesterol (mg/dL)	224.3 ± 39.1
CRP (μg/mL)	2.7 (1.4–5.7)
Caloric Intake (kcal/day)	1658.8 (1377.4–1957.8)
Lipid Intake (kcal/day) *	59.9 ± 18.6
RAE (mcg/day)	630.0 (447.3–973.6)
β-Carotene equivalents (mcg/day)	1785.0 (1325.7–2374.6)
Smoking (*n*, %)	
Never smokers	429 (83.8)
Current smokers	46 (9.0)
Former smokers	37 (7.2)
Cancer (*n*, %)	39 (7.6)

* Data are presented as number of cases (percentage), mean ± SD, or median and interquartile range.

**Table 2:** Relationship between retinol, pro-vitamin A and non-provitamin A carotenoids and Estradiol.

	Estradiol			
	Model 1 *		Model 2 **	
	β ± SE	*p* *	β ± SE	*p* **
Total carotenoids	−0.11 ± 0.05	0.03	−0.10 ± 0.03	0.005
Total pro-vitamin A carotenoids (μmol/L)	−0.07 ± 0.03	0.009	−0.21 ± 0.06	0.001
α-Carotene (μmol/L)	−0.010 ± 0.004	0.02	−1.59 ± 0.61	0.01
β-Carotene (μmol/L)	−0.076 ± 0.02	0.0007	−0.29 ± 0.08	0.0009
β-Cryptoxanthin (μmol/L)	0.010 ± 0.01	0.43	−0.11 ± 0.14	0.40
Total non-provitamin A carotenoids (μmol/L)	−0.038 ± 0.033	0.25	−0.09 ± 0.06	0.11
Lutein (μmol/L)	−0.012 ± 0.01	0.29	−0.28 ± 0.15	0.07
Zeaxanthin (μmol/L)	−0.0007 ± 0.002	0.70	−1.03 ± 0.97	0.28
Lycopene (μmol/L)	−0.024 ± 0.026	0.35	−0.07 ± 0.07	0.32
Retinol (μmol/L)	0.06 ± 0.04	0.11	0.05 ± 0.06	0.35

* Age adjusted; ** Adjusted for age, BMI, Testosterone, Insulin, Total-cholesterol, CRP, Smoking, AST, ALT, Creatinine, Cancer, Caloric Intake, Lipid Intake, RAE.

**Table 3:** Multivariate model addressing the relationship between α-Carotene and β-Carotene and Estradiol.

Estradiol		
Model 3		
	β ± SE	*p* *
α-Carotene (μmol/L)	−0.77 ± 0.70	0.26
β-Carotene (μmol/L)	−0.23 ± 0.09	0.01
BMI (kg/m^2^)	0.02 ± 0.005	<0.0001
Testosterone	0.49 ± 0.07	<0.0001
Insulin	−0.05 ± 0.04	0.22
Total Cholesterol	0.0006 ± 0.0006	0.26
CRP (μg/mL)	0.07 ± 0.02	0.002
Smoking	0.03 ± 0.04	0.34
AST	−0.001 ± 0.005	0.73
ALT	0.003 ± 0.003	0.39
Creatinine	0.15 ± 0.13	0.25
Cancer	−0.02 ± 0.08	0.73
Caloric Intake (kcal/day)	0.0001 ± 0.00009	0.29
Lipid Intake	−0.005 ± 0.002	0.03
RAE (mcg/day)	−0.00007 ± 0.00004	0.10

* Each line refers to a multivariate analysis including the covariates listed in the Table.

## 4. Discussion and Conclusions

In a cohort of late post-menopausal women not taking HRT, we found that serum β-carotene was independently and inversely associated with E2 levels. This is the first evidence of a relationship between retinol, carotenoids, and E2 levels in a representative cohort of late post-menopausal women with complete data on serum concentrations of the major dietary pro-vitamin and non-pro-vitamin A carotenoids and retinol.

Our results may be clinically relevant considering that the most common form of cancers in women are 17β-estradiol-responsive [28]. In particular, it is estimated that approximately 75% of breast cancer in postmenopausal population is estrogen-receptor-positive [29].

Interestingly, in spite of the significant and inverse association found between both β-carotene and α-carotene and E2 levels, only serum β-carotene was associated with E2 levels independently of α-carotene. On the basis of these findings, it can be speculated that there is a more prominent role for β-carotene than α-carotene in the modulation of E2 levels. However, we have to underline that experimental studies have particularly suggested an anti-estrogenic role for carotenoids exerted by the modulation of the biological activity of estradiol. However, given the nature of the study design we were not able to address this specific issue.

It is known that β-carotene represents the major provitamin A carotenoid in the Western diet [1]. To date, the only well-established pathophysiologic consequence of dietary carotenoid “deficiency” is considered the provitamin A activity of carotene, especially β-carotene [12]. Moreover, among the carotenoids, β-carotene was mainly involved in normal primary mammary epithelial cell differentiation and in the control of proliferation, suggesting a protective role against breast carcinogenesis [30].

In human mammary and endometrial cells, β-carotene inhibits the transactivation of estrogen response elements (ERE) induced by 17-β E2 [13]. Moreover, β-carotene at concentrations ranging from 0.25 µM to 10 µM significantly ameliorated catechol estrogen-mediated DNA damage *in vitro* [14].

However, it should be acknowledged that *in vitro* and *in vivo* studies support a role for retinol in counteracting estrogen signaling [7,8,9,10].

Retinoic acid has been shown to play a role in estrogen-responsive breast cancer cells by interfering with the activation of the lysine-specific demethylase 1LSD1 via the catalytic subunit of protein kinase A (PKA) [11]. In a recent study, Cheng and colleagues [7] demonstrated that retinoic acid inactivated local E2 production in the epithelial endometrial cells by augmenting the expression of 17β-hydroxysteroid dehydrogenase type 2, which is responsible for the rapid conversion of E2 into inactive estrone. Moreover, synthetic retinoid *N*-(4-hydroxyphenyl)-retinamide (4-HPR) also exhibited specific inhibitory actions on microsomal and cellular aromatase activity as well as the expression of aromatase mRNA induced by cAMP [10].

Surprisingly, we did not find any significant relationship between retinol and E2 levels. Interestingly, our data would also suggest that the potential β-carotene anti-estrogenic property is due to the intact molecule, rather than to intestinal cleavage products (retinoids) [2]. It is established that after an oral dose of β-carotene, both intact β-carotene and its metabolite retinol can be detected in the serum [2]. Interestingly, in human breast cancer cells, Elmi and colleagues documented an inhibitory action for synthetic eccentric cleavage products of β-carotene (the apo-beta-carotenoic acids: β-apo-14′-, β-apo-12′-, β-apo-10′- and β-apo-8′-CA) on estrogen receptor-positive cell growth. This activity was independent either of the conversion to all-trans-retinoic acid or the high-affinity binding to the retinoid acid receptors [31]. The authors underlined that the apo-β-carotenoic acids, though they are structurally similar to all-trans-retinoic acid, have different side chain lengths that might account for several independent activities [31].

Finally, the study did not find any significant association between each non-provitamin A carotenoid examined and E2. This is in contrast with the few existing data documenting a role for lycopene in the inhibition of estrogenic activity of 17 β-E2 in cancer cell cultures [13].

Given the limited studies on this topic, the underlying mechanisms contributing to the relationship between β-carotene and E2 need to be investigated. Several lines of evidence suggest that β-carotene might induce regulation of ER expression and antagonize estrogen binding to nuclear estrogen receptors [13]. However, other reports suggest that β-carotene does not necessarily play a role in estrogen-regulated pathways and its beneficial effect in hormone-dependent cancers might be realized through a steroid hormone-related mechanism, by diffusing through the cell membrane, serving as ligands for nuclear carotenoid-specific receptors (with or without first binding to cellular transport proteins) [32]. In MCF-7 cells, Nesaretnam *et al.* [32] showed that palm oil carotene concentrate containing 60% β-carotene, 30% α-carotene, and smaller amounts of lycopene, phytoene, and γ-carotene, causes a dose-dependent inhibition of E2-stimulated growth, but does not affect the expression of pS2 protein, which is closely regulated by E2.

Finally, in an attempt to understand if carotenoids could affect aromatase activity [9], we calculated the ratio between T and E2 as a proxy of the aromatase enzymatic activity. However, we failed to detect any significant association between carotenoids and the T/ E2 ratio.

### Limitations and Strengths

Given the cross-sectional design, a cause-effect relationship between pro-vitamin A carotenoids and E2 levels cannot be inferred and, therefore, the observed inverse relationship between β-carotene and E2 does not necessarily imply a causal pathway in the relationship between β-carotene and E2 in older women. Given the lack of epidemiological studies on this topic, it was difficult to compare our findings with other studies. Consequently, our results need to be confirmed in future investigations. Furthermore, because our study was based on a specific setting including only late post-menopausal women, the generalizability of our findings to adult individuals or to a different population (older US or similar populations with high supplements use) is unknown.

Moreover, no information was available on tissue E2 levels and estrone, which could have provided more details about the biological activity of estrogens.

Finally, we are aware that E2 and T levels were not measured by liquid chromatography tandem mass spectrometry (LC-MS/MS), which currently provides the most sensitive and best-validated method for the analysis of E2 and T in serum samples from post-menopausal women [33]. Notwithstanding these limitations, our study has multiple important strengths. This is the first attempt to provide novel insight into the relationship between multiple carotenoids, retinol, and E2 levels in a study cohort of late post-menopausal women with complete information on plasma concentrations of retinol, α-carotene, β-carotene, β-criptoxantin, lutein, zeaxanthin, and lycopene. This represents an important strength point of the study since blood carotenoid concentrations are more precise indicators of carotenoid status than the simple information collected by dietary questionnaires [24]. Furthermore, this study also provides a unique opportunity to address the relationship between physiologic levels of vitamin A/carotenoids and E2, as a very low percentage of subjects were using dietary supplements. This issue would be impossible to be tested using a US cohort where it has been estimated that about 40%–50% of participants are supplement users [34].

In addition, according to data from the Framingham Heart Study, we considered important confounders such as T, fasting insulin, total plasma cholesterol levels, BMI, and smoking status in our analysis [24].

Finally, participants were also carefully selected with respect to HRT status, and those taking HRT were excluded. However, participants used in the analysis were comparable with the HRT group for all the variables considered in the analysis presented here (data not shown).

In conclusion, in late post-menopausal women, plasma provitamin A carotenoid β-carotene levels were inversely associated with E2 independent of other carotenoids. The potential mechanisms underlying the modulation of E2 levels as well as the therapeutic role of these phyto-nutrients in estrogen-dependent cancer targets need to be evaluated in large-scale clinical trials.

## Abbreviations

T/E2, testosterone-to-estradiol ratio; ER, estrogen receptor; E2, 17 β-estradiol; T, Testosterone; AST, aspartate aminotransferase; ALT, alanine aminotransferase; CRP, C-reactive protein; MDC, minimum detectable concentration; CVs, coefficients of variation.
